# 

**Published:** 1910-01

**Authors:** 


### BOOKS RECEIVED.

Practical Study of Malaria. By William H. Deaderick, M.D., Member American Society of Tropical Medicine; Fellow London Society of Tropical Medicine and Hygiene. Octavo of 400 pages, with 100 illustrations, some in colors. Philadelphia and London: W. B. Saunders Co., 1909. (Cloth, $4.50 net.)

Surgery: Its Principles and Practice. In five volumes. By 66 eminent surgeons. Edited by W. W. Keen, M.D., LL.D., Hon. F.R.C.S., Eng. and Edin., Emeritus Professor of the Principles of Surgery and of Clinical Surgery, Jefferson Medical College, Phila. Volume V. Octavo of 1274 pages, with 550 illustrations, 45 in colors. Philadelphia and London: W. B. Saunders Company. 1909. (Per volume; cloth, $7.00; half morocco, $8.00, net prices.)

Report of the Commissioner of Education for the year ended June 30, 1909. Vol. I. Washington: Government Printing Office.

Transactions of the American Surgical Association. Vol. 27. Edited by Richard H. Harte, M.D., Recorder of the Association.

Transactions of the thirty-first annual meeting of the American Laryngological Association held at Boston, May 31, June 1, and 2, 1909. James E. Newcomb, M.D., Secretary.

Fifth annual report of the Henry Phipps Institute for the Study, Treatment and Prevention of Tuberculosis. February 1, 1907, to February 1, 1908. Edited by Joseph Walsh, A.M., M.D.

Dose Book and Prescription Writing. By E. Q. Thornton, M.D., Assistant Professor of Materia Medica, Jefferson Medical College, Philadelphia. Fourth edition revised. 12mo of 410 pages, illustrated. Philadelphia and London: W. B. Saunders Company, 1909. (Flexible leather, $2.00 net.)

A Manual of Normal Histology and Organography. By Charles Hill, Ph.D., M.D., Formerly Assistant Professor of Histology and Embryology, Northwestern University Medical School, Chicago. Second revised edition. 12mo of 468 pages, with 312 illustrations. Philadelphia and London: W. B. Saunders Company, 1909. (Flexible leather, $2.00 net.)

A Textbook of Physiology: for medical students and physicians. By William H. Howell, Ph.D., M.D., LL.D., Professor of Physiology, Johns Hopkins University, Baltimore. Third edition. Revised. Octavo of 998 pages, illustrated. Philadelphia and London: W. B. Saunders Company, 1909. (Cloth, $4.00; half morocco, $5.50 net prices.)

A Textbook upon the Pathogenic Bacteria. For students of medicine and physicians. By Joseph McFarland, M.D., Professor of Pathology and Bacteriology in the Medico-Chirurgical College, Philadelphia. Sixth revised edition. Octavo of 709 pages, illustrated, a number in colors. Philadelphia and London: W. B. Saunders Company, 1909. (Cloth, $3.50 net.)

A Textbook on the Practice of Gynecology. For practitioners and students. By W. Easterly Ashton, M.D., LL.D., Professor of Gynecology in the Medico-Chirurgical College of Philadelphia. Fourth edition, revised. Octavo of 1099 pages, with 1058 original line drawings. Philadelphia and London: W. B. Saunders Company, 1909. (Cloth, $6.50; half morocco, $8.00, net prices.)

Primer of Sanitation. Disease Germs and How to Fight Them. By John W. Ritchie, Professor of Biology, College of William and Mary, Virginia. 12mo, pp. 206. Illustrated by Karl Hassmann. Yonkers: World Book Co. (Cloth, 50 cents.)

A Textbook of the Practice of Medicine. By James M. Anders, M.D., Ph.D., LL.D., Professor of the Theory and Practice of Medicine and of Clinical Medicine, Medico-Chirurgical College, Philadelphia. Ninth revised edition. Octavo of 1326 pages, fully illustrated. Philadelphia and London: W. B. Saunders Company, 1909. (Cloth, $5.50; half morocco, $7.00 net prices.)

A Practical Treatise on Ophthalomology. By L. Webster Fox, M.D., Professor of Ophthalmology in the Medico-Chirurgical College, Philadelphia. Octavo, pp. 832. Illustrated with 6 colored plates and 300 illustrations in the text. New York and London: D. Appleton & Co. 1910. (Cloth, $6.00.)

Modern Clinical Medicine. Diseases of Children. Edited by Abraham Jacobi, M.D., Consulting Physician to Mt. Sinai, Bellevue, Babies' and other hospitals, New York. Translated from "Die Deutsche Klinik" under the general editorial charge of Julius L. Salinger, M.D. Octavo, pp. 843. Illustrated. New York and London: D. Appleton & Co. 1910. (Cloth, $6.00.)

Index of the Progress Medical. Announcement number for students and practitioners. Thirty-seventh year. 1909-1910.

Transactions of the Section on Obstetrics and Diseases of Women of the American Medical Association at the Sixtieth Annual Session, held at Atlantic City, N. J., June 8 to 11, 1909. C. Jeff Miller, M.D., New Orleans, Secretary.

Progressive Medicine, Vol. XI, December, 1909. A Quarterly Digest of Advances, Discoveries and Improvements in the Medical and Surgical Sciences. Edited by Hobart Amory Hare, M.D., Professor of Therapeutics and Materia Medica in the Jefferson Medical College of Philadelphia. Octavo, 334 pages. Illustrated. Lea & Febiger, Publishers, Philadelphia and New York. (Per annum, in four cloth-bound volumes, $9.00; in paper binding, $6.00, carriage paid to any address.)

The Practical Medicine Series. Ten volumes. Under the general editorial charge of Gustavus P. Head, M.D., Professor of Laryngology and Rhinology in the Chicago Post-Graduate Medical School. Vol. IX. Skin and Venereal Diseases and Miscellaneous Topics. Edited by W. L. Baum, M.D., and Harold N. Moyer, M.D., Series 1909. Chicago. The Year Book Publishers. (Price, $1.25; entire series, $10.00.)

